# The Biocontrol Potential of Endophytic *Trichoderma* Fungi Isolated from Hungarian Grapevines. Part I. Isolation, Identification and In Vitro Studies

**DOI:** 10.3390/pathogens10121612

**Published:** 2021-12-10

**Authors:** Csilla Kovács, András Csótó, Károly Pál, Antal Nagy, Erzsébet Fekete, Levente Karaffa, Christian P. Kubicek, Erzsébet Sándor

**Affiliations:** 1 Research Institute Újfehértó, Agricultural Research and Educational Farm, University of Debrecen, Vadas tag 2, H-4244 Újfehértó, Hungary; kovacs.csilla@agr.unideb.hu; 2 Institute of Plant Protection, Faculty of Agricultural and Food Science and Environmental Management, University of Debrecen, Böszörményi út 138, H-4032 Debrecen, Hungary; csoto.andras@agr.unideb.hu (A.Cs.); nagyanti@agr.unideb.hu (A.N.); 3 Kálmán Kerpely Doctoral School, University of Debrecen, Böszörményi út 138, H-4032 Debrecen, Hungary; 4 Institute of Food Science, Faculty of Agricultural and Food Science and Environmental Management, University of Debrecen, Böszörményi út 138, H-4032 Debrecen, Hungary; pal.karoly@agr.unideb.hu; 5 Department of Biochemical Engineering, Faculty of Science and Technology, University of Debrecen, Egyetem tér 1, H-4032 Debrecen, Hungary; kicsizsoka@yahoo.com (E.F.); levente.karaffa@science.unideb.hu (L.K.); 6 Institute of Metagenomics, University of Debrecen, Egyetem tér 1, H-4032 Debrecen, Hungary; 7 Institute of Chemical, Environmental & Bioscience Engineering, TU Wien, Vienna A-1060, Austria; peter.kubicek@tuwien.ac.at

**Keywords:** *Vitis vinifera*, *Trichoderma afroharzianum*, *Trichoderma simmonsii*, mycelial growth, dual plate tests, fungicide tolerance

## Abstract

This paper reports on the identification and in vitro characterization of several *Trichoderma* strains isolated from the Tokaj Wine Region in North-East Hungary. Ten isolates were analyzed and found to consist of six individual species—*T. gamsii*, *T. orientale*, *T. simmonsii*, *T. afroharzianum*, *T. atrobrunneum* and *T. harzianum* *sensu stricto*. The growth potential of the strains was assessed at a range of temperatures. We also report here on the in vitro biocontrol properties and fungicide tolerance of the most promising strains.

## 1. Introduction

Interest in non-chemical pesticides is increasing worldwide. Consumers’ requirements for lower pesticide residues and chemical pollutant levels, as well as the emergence of resistance among pests has resulted in a worldwide tendency towards restricting the use of chemical pesticides and searching for alternative control methods. For example, the recent Farm to Fork strategy of the European Green Deal requires a 50% reduction of the use of chemical pesticides by 2030 within the European Union. Biopesticides are amongst the most promising agents to replace chemical pesticides. However, they should not only be effective against plant pathogens, but need to fulfill economic and safety criteria as well [1].

Mycoparasitic and antagonistic properties of species of the fungal genus *Trichoderma* have been known for a long time [2]. While mycoparasitism on basidiomycetes is a general property of the *Hypocreaceae*, *Trichoderma* is special because its mycoparasitism also extends to ascomycetes and even other *Trichoderma* spp. [3]. In fact, the first report on the isolation of a species within this genus [4] roughly coincides with describing their mycoparasitic effect against *Fusarium oxysporum* [5] and their role in woody disease control [6]. *Trichoderma* (*Hypocrea*) species are opportunistic colonizers of various habitats, and while most species are found on the basidiocarps of basidiomycetes, they can be isolated from soil and plants, as well [7]. The arsenal *Trichoderma* are able to deploy to prevent infection and damage by the plant pathogens is diverse: they can prevent the germination of spores of plant pathogenic fungi by producing secondary metabolites and are able to destroy them with their secreted cell wall-degrading enzymes. Some *Trichoderma* strains also have been proven to increase resistance towards pathogens by inducing systemic and local defense of the plant. Furthermore, their promotion of plant growth, development, and nutrient uptake are also reported [8,9,10,11]. *Trichoderma* can utilize all sorts of nutrients and therefore its conidia can be produced en masse on cheap growth substrates such as agro-industrial wastes [12].

Currently, genus *Trichoderma* contains some 400 accepted species. Due to inter-species similarities, accurate identification based solely on morphological characteristics is not possible [13,14,15]. Therefore *Trichoderma* spp. described in the last 15 years have been identified by means of phylogenetic analyses of DNA sequence data [16].

Although reports on *Trichoderma* strains with promising biopesticide characteristics are widespread [17,18,19,20,21], and many products associated with *Trichoderma* are listed on databases [22,23], only a handful of strains from seven species are distributed in the single US and EU markets (Table 1). It must be noted though that in certain countries some strains are available via local distributors (e.g., *Trichoderma atrobrunneum* ITEM 908 in Italy by Agrifutur Inc., Alfianello, Italy). On the other hand, some previously marketed products such as Trichodex (Makhteshim Chemical Works Ltd., Beer-Sheva, Israel) containing *T. harzianum sensu stricto* Rifai T-39 has been withdrawn from the market [24,25]. Valid taxonomical status has changed several times following the description and registration of the marketed *Trichoderma* strains, particularly in the case of those originally defined as *Trichoderma harzianum* Rifai [26]. In most cases, marker sequences or even full genome sequences (*Trichoderma atrobrunneum* ITEM 908 and *Trichoderma afroharzianum* T-22) are available today for correct identification (Table 1).

Because of their opportunistic nature, some *Trichoderma* species can occur in the rhizosphere, permanently colonizing the root tissues [2,27], while others are facultative endophytes of aerial plant tissues [28,29,30]. Reports on endophytic strains from grapevine are rare, and they are scarcely ever characterized or identified on the species level. *Trichoderma* sp. was reported to be present in debarked young grapevine in Switzerland [31]. In a comprehensive study from Spain, 44 endophytic *Trichoderma* strains were found among 585 endophytic fungi isolated from different grapevine cultivars [32]. Jayawardena et al. [33] described three *Trichoderma* species (putatively identified as “*T. atroviride*”, “*T.* cf. *harzianum*” and “*T. lixii*” by ITS sequencing), isolated from *Vitis vinifera* from China, but they were considered of saprophytic origin. More recently, Silva-Valderrama et al. [21] studied a grapevine endophyte *Trichoderma* sp. isolated earlier in Chile. Unfortunately, the *Trichoderma* species identification in all these studies was based solely on the ITS1 and ITS2 containing rRNA regions, and the sequences were not compared to those of the ex-type strain and therefore species identity is doubtful. Carro-Huerga [18] described an endophytic *Trichoderma* sp. from Spain, which—based on a multigene analysis—clustered within the *T. harzianum* species clade. Whether it is a member of one of the already described species in this clade or a new species could not be decided, however, because only single strains from each species were used for the construction of the phylogenetic tree.

In this paper, in vitro characterization of several *Trichoderma* isolates collected from the Tokaj Wine Region in Hungary is reported. This is the first report on properly identified and characterized endophytic *Trichoderma* strains from Europe. The strains were found during the screening of a local vineyard for grapevine trunk diseases (GTDs) pathogens and were identified on the basis of ITS1 and ITS2 containing rRNA region and *tef1* sequences. The growth potential of the strains was assessed at a range of temperatures. Potential human pathogens—based on temperature preferences and taxonomic characteristics—were discarded. We also report here on the in vitro biocontrol properties and fungicide tolerance of the most promising strains.

## 2. Materials and Methods

### 2.1. Isolation

**Figure 1:**
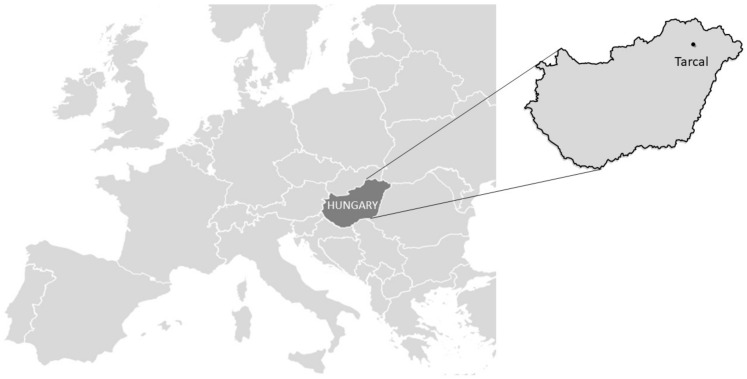
The origin of the *Trichoderma* strains. Tarcal, Tokaj Wine Region, Hungary.

Ten *Trichoderma* strains were isolated (TR01-TR10) from ‘Furmint’ cultivar of grapevine (*Vitis vinifera*, L.) from the Tokaj Wine Region, Hungary (Figure 1) in 2014. The 22 years old vineyard showed very high (>34%) occurrence of the grapevine trunk diseases symptoms (GTDs) [34]. Wood chips from cordon of grapevine plants with no GTD symptom expressions were debarked and surface sterilized, and chips were placed on malt extract agar medium (MEA, Scharlau, Barcelona, Spain) in Petri dishes with a sterile scalpel, under aseptic conditions following the method described by Kovács et al. [34]. Emerging *Trichoderma*-like colonies were then transferred to new 2% MEA plates and isolates were purified as described in Kulling et al. [35]. Conidial suspensions were stored in 40% glycerol at −80 °C in the Laboratory of Microbiological Group, Institute of Food Science, University of Debrecen. TR04, TR05 and TR08 strains have also been deposited in the National Collection of Agricultural and Industrial Microorganism (NCAIM, Budapest, Hungary) as NCAIM (P) F001456, NCAIM (P) F001457 and NCAIM (P) F001458.

### 2.2. Identification of Endophytic Trichoderma Species

*Trichoderma* species were first roughly attributed to various species clades on the basis of their ITS1 and ITS2 containing rRNA sequences ([36], primers: SR6R and LR1; Table 2). Their species identity was then assessed by sequencing the large intron of the *tef1* gene encoding translation elongation factor 1α ([37], primers: EF1 728F and EF1 986R). In case of 100% sequence identity with the ex-type strain of a known species, the isolate was considered to belong to this species. If the sequence of the isolate was not 100% identical to any of the known species from this clade, a phylogenetic tree was constructed using the ex-type strains of the species with closest similarity, and other isolates of them whose species identity had been approved [7,26].

To this end, DNA was isolated from the monosporic *Trichoderma* strains with disrupting the 3 days old fungal mycelia grown on potato dextrose agar (PDA, Scharlau, Barcelona, Spain) at 25 °C in dark with MagNALyser (Roche, Mannheim, Germany). DNA isolation was carried out using NucleoSpin Plant II kit (Macherey-Nagel, Düren, Germany) according to the manufacturer’s instructions. DNA concentrations were determined by a NanoDrop 2000 spectrophotometer (Thermo Scientific, Wilmington, DE, USA). The isolated DNA was checked up on 0.8% agarose gel in TAE buffer.

DNA regions containing the ITS1 and ITS2 locus and the *tef1* fragment were amplified with different universal primers (Table 2) in MyGenie 96 Gradient Thermal Block thermal cycler (Bioneer, Daejeon, South Korea) with the PCR program described in Váczy et al. [38]. The annealing temperature applied as well as the primers used for the amplification are summarized in Table 2.

NucleoSpin Gel and PCR Clean-up kit (Macherey-Nagel) was used for the purification of PCR products. DNA concentrations measurements were performed by NanoDrop 2000 (Thermo Scientific) from 2 µL DNA. Sequencing of the purified amplification products was performed by Microsynth Austria GmbH (Vienna, Austria). Sequences were deposited in GenBank (OK560824-OK560833 and OK655885-OK655894).

The sequences were first compared with those deposited in the NCBI GenBank database by NBLAST analysis [39]. For phylogenetic analysis these were aligned with Clustal-X [40,41,42] and manually checked for ambiguities and adjusted, when necessary, using Genedoc [43]. Phylogenetic analysis was performed with MEGA 7.0 program [44]. The maximum likelihood method, based on the JC model was used for the ITS1 and ITS2 containing rRNA region and K2P model for the *tef1* sequences. Positions containing gaps and missing data were not considered. For maximum likelihood analyses, the nearest-neighbor interchange was used as the heuristic method for tree inference. Support for internal branches was assessed by 1000 bootstrapped pseudoreplicates of data.

### 2.3. Mycelial Growth

The mycelial growth of the *Trichoderma* isolates was determined at different temperatures (5; 18.5; 20; 22.5; 25; 30 and 37 °C) in three replicates. A 10 mm diameter mycelial plug was cut from the growing edge of the fungal colonies and placed on the center of potato dextrose agar (PDA, Scharlau, Barcelona, Spain) in a 90 mm diameter Petri-dish. Two colony diameters were measured regularly for 4 days, or until the colonies reached the edge of the Petri-dish. Average data calculated from the two colony diameters were used for further statistical analyses.

The mycelial growth was evaluated separately at 5 °C, at room temperature or at typical field condition by data measured between 18.5 and 25 °C and at 37 °C. Comparison of species and strains were carried out at two times at the start of the intensive growth and before the fastest-growing strain reached the edge of the Petri-dish.

The fit of our data to the assumptions of parametric tests was tested by Levene test and Q-Q plots. Since the data did not fulfill the assumptions, the growth potential of the species and strains were compared with Kruskall-Wallis (K-W) nonparametric test. If this showed significant differences the post-hoc comparisons were made by Mann-Whitney U (M-W) test. As well, this latter test was used when two independent groups were analyzed.

### 2.4. Biocontrol Index of Different Trichoderma Species

The mycoparasitic ability of *Trichoderma* isolates was studied according to the method of [45], by BCI (biocontrol index) determination as follows:

(1) $$\mathrm{BCI}\;\left(\%\right)=\left(\frac{d_{A}}{\left(d_{A}+d_{P}\right)}\right)\times 100$$

where d_A_: horizontal diameter of mycelial growth of the antagonist on PDA; d_P_: horizontal diameter of mycelial growth of the pathogen on PDA.

The tested pathogens were previously isolated and identified in the University of Debrecen Microbiological Laboratory of Food Science Institute (Debrecen, Hungary). *Aphanomyces cochlioides* and *Pythium acantophoron* were purchased from the Westerdijk Fungal Biodiversity Institute (Utrecht, The Netherlands). The plant pathogens used in the BCI tests are summarized in Table 3.

Two days old *Trichoderma* and three days old pathogen colonies grown on PDA medium were used for inoculation. A plant pathogen hyphal plug was first inoculated 1.5 cm away from the center of a Petri-dish, as described above for mycelial growth test. Following a 24 h long incubation, the tested *Trichoderma* mycelia plug was also inoculated 3 cm away from the pathogen on the same plate. Pictures were taken following 10 days incubation at 25 °C in the dark. Experiments were carried out in triplicates. Samples from the interacting zones were prepared and were screened for loops, using an AxioImager light microscope (Zeiss, Oberkochen, Germany).

### 2.5. Fungicide Tolerance Test

Some of the fungicides routinely used in Hungarian vineyards were utilized to test resistance of the isolated *Trichoderma* strains (Table 4). We used the highest recommended concentration to be applied in vineyards, calculated from the product labels (Table 4).

Fungicides were added to the still-fluid PDA after it cooled down to 50 °C following sterilization, just before being poured into Petri-dishes, thereby avoiding heat-degradation of the chemicals. Inoculation was performed as described for the mycelial growth tests, while incubation occurred at 25 °C in the dark for 54 h. Growth inhibition was calculated from three replicates by comparing to the mycelial growth on fungicide free PDA, as follows:

(2) $$\mathrm{Growth}\;\mathrm{inhibition}\;\left(\%\right)=\left(\frac{d_{c}-d_{f}}{d_{c}}\right)\times 100$$

where d_f_: diameter of mycelial growth on PDA containing fungicide; d_c_: diameter of mycelial growth on PDA.

## 3. Results

### 3.1. Strain Identification

**Figure 2:**
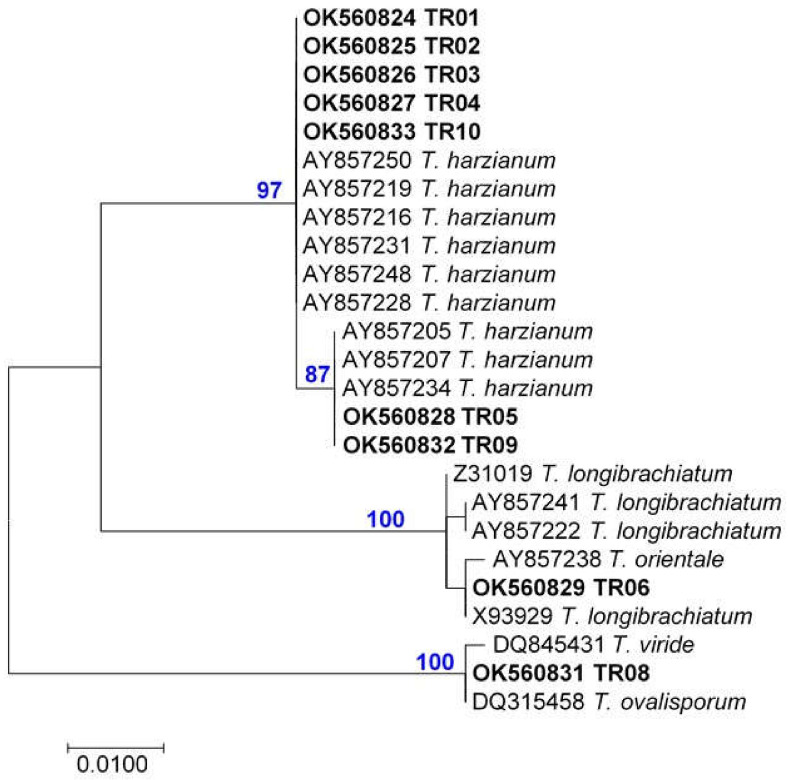
Greatest log likelihood ITS Maximum Parsimony phylogenetic tree generated from TR01-TR10 (Table 5) and deponated sequences with Accession Number before species name. The length of branches is proportional to the number of nucleotide differences in the sequences, the scale is under the dendrogram. The numbers above branches show the results of the bootstrap analysis values from 1000 replicates.

**Figure 3:**
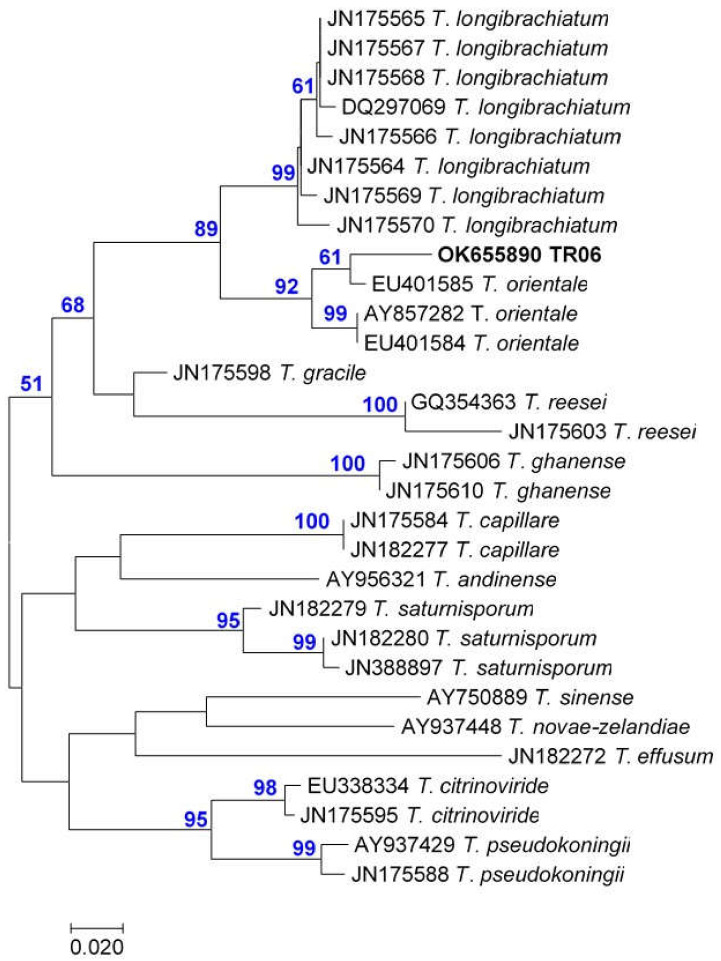
Maximum Parsimony phylogenetic tree generated from *tef1* of *Trichoderma* isolates TR01–TR10 (Table 5) and deponated sequences with Accession Number before species name. The length of branches is proportional to the number of nucleotide differences in the sequences, the scale is under the dendrogram. The numbers above branches show the results of the bootstrap analysis values higher than 50, from 1000 replicates.

The phylogenetic analysis of the ITS sequences placed eight of the ten endophytic *Trichoderma* isolates (TR01-05, TR07 and TR09-10) into the Harzianum species clade of *Trichoderma* (Figure 2). Only two strains belonged to other groups. The TR08 clustered with the species of the Viride clade of *Trichoderma* and was identified through *tef1* sequence as *Trichoderma gamsii* (Table 5). The TR06 clustered with species from the Longibrachiatum clade of *Trichoderma*, and the phylogenetic analysis of its *tef1* sequence identified this strain as *Trichoderma orientale* (Figure 3, Table 5).

The isolates that fell based on ITS1 and 2 sequence analysis into the Harzianum species clade, consisted of several species: four of the eight strains were identified as *Trichoderma simmonsii*, two as *T. harzianum sensu stricto*, and one each as *Trichoderma afroharzianum*, and *Trichoderma atrobrunneum* (Table 5).

Molecular phylogenetic analysis by maximum likelihood method (Kimura 2-parameter model. Initial tree(s) for the heuristic search were obtained automatically by applying Neighbor-Join and BioNJ algorithms to a matrix of pairwise distances estimated using the maximum composite likelihood (MCL) approach, and then selecting the topology with superior log likelihood value. All positions containing gaps and missing data were eliminated. Evolutionary analyses were conducted in MEGA7.

### 3.2. Growth Characteristics at Different Temperatures

**Figure 4:**
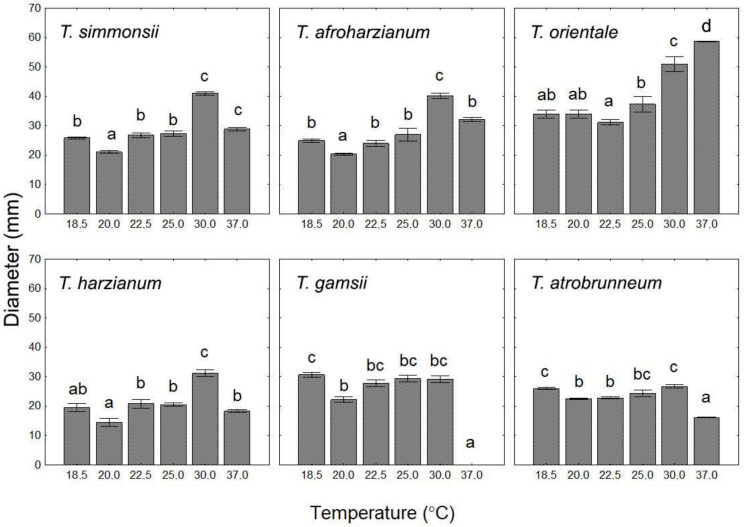
Average mycelial growth of endophytic *Trichoderma* species isolated in the Tokaj Wine Region, Hungary. Colony diameter was measured at 30 h following inoculation. Different letters show significant differences based on pairwise comparison with Mann-Whitney U test (*p* < 0.05).

The mycelial growth of the different isolates was tested in vitro. Rapid growth was detected on PDA for all strains: they overgrew the entire Petri-dish within one week at temperatures between 18.5 and 30 °C (Figure 4). However, at 5 °C, mycelial growth was detected only in the second week (Table 6a). The majority of the strains grew fastest at 30 °C, except for TR06 (*T. orientale*) and TR08 (*T. gamsii*), whose growth maxima occurred at 37 °C and 25 °C (Figure 4).

The majority of the strains grew faster at higher temperatures. *T. simmonsii* (TR01-03 and TR05) showed fastest growth at 30 °C. The optimal growth temperature of *T. afroharzianum* (TR04) and *T. harzianum* (TR07 and TR10) was 30 °C as well. *T. atrobrunneum* (TR09) and *T. gamsii* (TR08) showed two different optima. The former grew faster at 18, 25 and 30 °C, while the latter grew similarly at 18, 22.5, 25 and 30 °C and did not grow at 37 °C. Only *T. orientale* (TR06) preferred 37 °C (i.e., the highest temperature tested) and showed significantly faster growth under that condition (Figure 4).

Growth characteristics changed during the cultivation, therefore data from the first day following the appearance of mycelia (24 h at 18.5–37 °C, and 168 h at 5 °C) were excluded from further analysis.

Although all strains of the six species have started to grow at 5 °C after one week, there were marked differences (K-W: H(5, 30) = 21.03, *p* = 0.0008 at 7th day, and = 21.91, *p* = 0.0005 at 14th day) between them (Table 6a). The fastest growth was detected for *T. simmonsii, T. afroharzianum* and *T. atrobrunneum*, while *T. harzianum sensu stricto* was the slowest both at the early (8th day) and the late (14th day) stages. The growth of *T. gamsii* (TR06) has changed over time; it was one of the slowest in the first 8 days, similarly to *T. harzianum sensu stricto*, but later showed accelerated growth, similar to *T. orientale* (TR08). Surprisingly, there was marked differences between the strains of the *T. simmonsii* although they were collected from the same vineyard, similarly to the two *T. harzianum sensu stricto* strains.

The mycelial growth of the different species and strains had significant differences (K-W: H (5, 120) = 64.86 *p* < 0.0001 at 24 h, and = 29.716 *p* < 0.0001 at 54 h) at the temperature range between 18.5 °C and 25 °C (room temperature as well as typical field conditions). At this temperature range, the growth of *T. harzianum sensu stricto* was the slowest, similarly to the case at 5 °C. The fastest growing isolate was *T. orientale*, followed by *T. gamsii*. The growth of *T. simmonsii* and *T. afroharzianum* fell between the fastest and the slowest ones and were quite similar to each other. *T. atrobrunneum* was growing as slowly as *T. harzianum sensu stricto* at 54 h (Table 6b).

The strains belonging to *T. simmonsii* (K-W: H(3, 48) = 4.48, *p* = 0.21 at 24 h, and = 4.65 *p* = 0.1993 at 54 h) and *T. harzianum sensu stricto* (M-W: U = 65.00, *p* = 0.68, at 24 h, and U = 70.00, *p* = 0.91 at 54 h) had similar characteristics at this temperature range (Table 6b).

The growth potential at 37 °C was tested to select potential human pathogens. *T. orientale* showed the highest growth rate at that temperature (Figure 4), and its initial growth rate was significantly higher than that of the other strains (Table 6c). Growth was not detected at this temperature for *T. gamsii* (Figure 4, Table 6c).

Although *T. orientale* (TR06) showed the best growth characteristics of all endophytic *Trichoderma* strains, it was excluded from further analysis due to its rapid growth at 37 °C. *T. harzianum sensu stricto* had the worse growth potential at each temperature tested except for 37 °C. Although *T. gamsii* did not growth at 37 °C, it was one of the slowest growing isolate at 5 °C as well. As a result of the growth tests, the two strains TR05 and TR04 were chosen for further analysis. They belong to two different species within the Harzianum clade: *T. simmonsii* and *T. afroharzianum*. They showed excellent growth both at 5 °C, and in a range that covers the most typical field conditions (18.5–25 °C), but their growth was significantly slower than that of the potential human pathogen *T. orientale.*

### 3.3. Potential for Biocontrol

**Figure 5:**
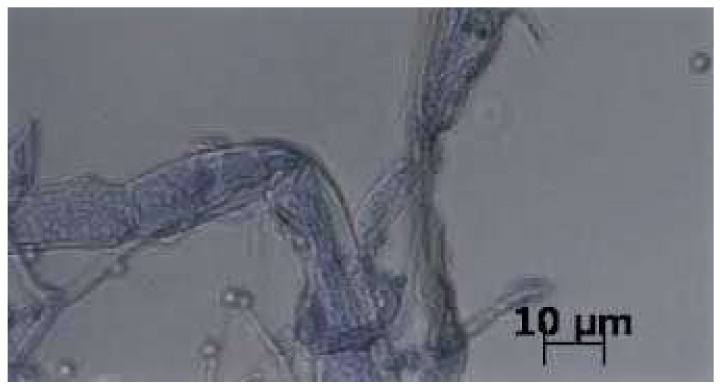
Hyphal coiling of *Trichoderma afroharzianum* (TR04 strain) against *Diplodia seriata* hypha. Sample was stained with lactophenol cotton blue. Images were prepared with Zeiss AxioImager phase-contrast microscope, equipped with AxioCam MRc5 camera.

Biocontrol potential in dual culture tests were studied for the TR04 and TR05 strains, against different fungi with pathogenic potential against plants. The TR04 (*T. afroharzianum*) showed high BCI against all the tested Ascomycota and Oomycota pathogens (Table 7), including three GTD pathogens isolated from grapevine (*Diplodia seriata*, *Eutypa lata*, *Neofusicoccum parvum*). This strain completely overgrew all but two pathogens and sporulated on their mycelia, killing the pathogen colonies. The TR05 strain also had high BCI (>80%), except against *Botryosphaeria dothidea*. Mycoparasitic activity was also detected by hyphal coiling and penetration (Figure 5).

### 3.4. Pesticide Tolerance

None of the tested pesticides could completely inhibit the growth of the *Trichoderma* TR04 and TR05 strains (Table 8). Three of the pesticides (Orvego, Sercadis, Teldor 500 SC) did not, or only slightly (7%) inhibited the mycelial growth of the TR04 (*T. afroharzianum*) and TR05 (*T. simmonsii*) strains in the concentrations used in vineyards. Only Talentum 20 EW could inhibit the growth of both strains in >50%—in this case, the concentration of the active ingredient (myclobutanil) in the growth medium was 80 mg/L. In case of Rally Q SC (45 mg/L myclobutanil concentration), inhibition fell below 50% for both strains, despite that this pesticide contains an additional active ingredient (Quinoxifen), too.

## 4. Discussion

Sustainable, environmental-friendly and climate neutral agricultural production is among the most important goals to be implemented all across the world. In the European Union the most recent agronomical frame for legislation, the Farm to Fork Strategy of the European Green Deal [46] has stated that “*The EU needs to develop innovative ways to protect harvests from pests and diseases and to consider the potential role of new innovative techniques to improve the sustainability of the food system, while ensuring that they are safe*”. The aim is to replace 50% of the chemical pesticides with biological, physical and other non-chemical methods in the integrated pest management within a decade. Therefore, appropriate pest control methods such as antagonist or hyper-parasitic (micro)organisms must be provided on the emerging fields of organic farming [47]. Their advantages include the lack of resistance developing against them, their ability to adapt to the evolving pests, lower toxicity, faster decomposition rate, and consequently the lack of remnant hazardous residues. Negative impact on environment, human or animal health is minimized, particularly if their environment of origin is similar to the one in which they are supposed to be used [1,48].

One of the fundamental requirements of any mycopesticide is proper identification and characterization [49]. Identification is also required to detect the relationship to known plant, animal or human pathogens.

*Trichoderma* spp. are among the most commonly marketed and employed microbial agents in agriculture, used also as biofertilizer and biostimulant in addition to being a biopesticide. Their efficiency is due to: (i) efficient competition for nutrients and space competitors, (ii) their ability to eliminate plant pathogen fungi via direct interaction (such as production of antimicrobial substances, penetration and others), and (iii) their ability to induce systemic plant resistance [50]. Although quite a few registered *Trichoderma*-containing products are available commercially, the majority of them are restricted to a single or a handful of countries [24]. Several of these products contain undefined strains and could potentially include pathogens of cultivated mushrooms or facultative pathogens of immunocompromised mammals including human [51]. *Trichoderma* are also occasionally part of a mixture of ingredients [24]. Although almost all registered and marketed *Trichoderma* contain isolates from the soil, there are reports about root, wood or leaf endophytes as well [21,52,53,54,55].

The ten endophytic *Trichoderma* strains were isolated and identified from grapevine cordon wood in an old vineyard in the Tokaj Vine Region, Hungary (Table 5). They were identified as species belonging to three large clades: Harzianum, Viride and Longibrachiatum. One strain (TR06) belonging to the Longibrachiatum clade of *Trichoderma*, was identified as *T. orientale* (Table 5, Figure 3). This species was described first as *Hypocrea orientalis* in 1998 [26] and has a global distribution [56]. It grows on wood and in soil [57]. *T. orientale* and the closely related, sympatric *T. longibrachiatum* are well known as opportunistic pathogens of immunocompromised human and animal [7,51,58]. Also in this study, the TR06 *T. orientale* strain was shown to grow the fastest at 37 °C, thus it was excluded from further studies.

The Viride clade of *Trichoderma* was also represented by one strain (TR08), identified as *T. gamsii* based on its ITS and *tef1* sequences. It was described by Samuels and Druzhinina [14] as a close relative of *T. viride* and *T. atroviride* [13,59]. *Trichoderma gamsii* can be isolated from many different geographical locations and is also known as an endophyte of the traditional Chinese medicinal plant *Panax notoginseng* [60]. This species can be found in registered, commercially available biocontrol products (Table 1, [24]). The isolated *T. gamsii* strain displayed reduced growth at low temperature (5 °C), limiting its further application. It should be noted that low temperature growth tests are rarely performed during the characterization *Trichoderma* species. Since it may take longer periods of time to detect growth at low temperatures, limited cultivation time may cause false negative results [19]. Some biocontrol *Trichoderma* strains isolated from soil (putatively identified as “*T. aureoviride*, *T. harzianum* and *T. viride*”) were reported to grow well at 5 °C [51].

Eight of the isolated endophytic strains were identified within the Harzianum clade. Only two strains were identified as *T. harzianum sensu stricto* (TR07 and TR10), and they displayed the poorest growth potential (Table 6). The most widely available biocontrol strain, “*T. harzianum* T22”, which is the active ingredient in several biocontrol and plant growth-stimulating products (Table 1, [61]), is in fact not *T. harzianum sensu stricto* but *T. afroharzianum* [26]. First found in Africa, it is now believed to be ubiquitous, both in the soil and on roots [62], and grows well at 35 °C. It was represented by one strain (TR04) among our grapevine endophytic isolates with excellent growth potential even at 5 °C.

*T. atrobrunneum* was also represented with one strain (TR09) in our isolates, with growth characteristics being similar to *T. afroharzianum*, except at 30 °C where the growth of *T. atrobrunneum* was slower. These species are known only from temperate regions. *T. simmonsii* was first isolated from rotting bark in the United States in 1991 [26]. It most commonly occurs on rotting woody parts and has already been isolated in many European countries and various parts of the United States. It is marked as Trichosan^®^ and Vitalin T-50^®^ [26]. This species was represented by four isolates (40%, the highest percentage) in our study.

Two strains (TR04 and TR05) were chosen for further studies based on the criteria of good growth at 5 °C, as well as at ambient temperature range, but with limited growth at 37 °C. They both exhibited excellent BCI against the tested GTD pathogens isolated from grapevine (*Diplodia seriata*, *Eutypa lata*, *Neofusicoccum parvum*). Their BCIs were also good towards Ooomycota and Ascomycota plant pathogens isolated from other hosts.

The isolated strains with appropriate growth characteristics and good BCI were tested for potential resistance against fungicides routinely applied in vineyards. None of the tested fungicides could completely inhibit the growth of the two most promising strains (TR04, TR05). It was not surprising that the pesticide used against downy mildew (Orvego)—whose active ingredients are dimethomorph (targeting cellulose synthase) and ametoctradin (affecting respiration as a Qo site inhibitor)—did not inhibit mycelial growth of either of the two *Trichoderma* strains. It was more of a surprise that the strains were also insensitive towards both the fluxapyroxad- and the fenhexamid-containing pesticides. Fluxapyroxad is a succinate dehydrogenase inhibitor affecting respiration, while fenhexamid is a keto reductase inhibitor within sterol biosynthesis [63]. Even a powerful pesticide (Rally Q SC) with combined myclobutanil (demethylation inhibitor in sterol biosynthesis) and quinoxyfen (with signal transduction mode of action) content showed <50% inhibition over mycelial growth. Although the primary reason to use biocontrol agents such as *Trichoderma* is to reduce the usage of chemical pesticides, applying fungicide-tolerant strains within a complex integrated plant protection strategy (IPM) is as well lucrative. Several studies have reported resistance towards a variety of fungicides [64,65,66,67,68,69,70,71,72] and fenhexamid tolerance of a putative *T. harzianum* strain (MAUL-20) has also been reported [69,73]. However, myclobutanil tolerance of our isolates was much higher than previously reported for *Trichoderma* [74].

*Trichoderma* strains isolated from healthy plants from agricultural fields may have several advantages. They have already adapted to the host, the particular agricultural practices (e.g., fungicide application) as well as to the climatic conditions (e.g., cold tolerance). Moreover, endophytic strains—whose growth is preferred *in planta*—may pose less harm to the soil microbiome. In summary, we can conclude that based on in vitro studies, the endophytic *T. afroharzianum* and *T. simmonsii* strains isolated from a Hungarian vineyard are promising biocontrol agents, and their potential clearly warrants further *in planta* and *in field* studies. These are described and discussed in a subsequent paper of this *Pathogens* Special Issue.

## Figures and Tables

**Table 1:** *Trichoderma* species in marketed biocontrol products.

*Trichoderma* Species	Strain	Deponated Sequences/Genome	Product	Producer/Distributor	Reference/Database Information
Harzianum clade of *Trichoderma*					
*T. afroharzianum* (formerly: *T. harzianum Rifai*)	T-22 (KRL-AG2, ATCC20847)	https://genome.jgi.doe.gov/portal/pages/projectStatus.jsf?db=TriharT22_1 (accessed on 7 December 2021)	T-22 WP BW240 G ^a^ (PlantShield^®^ HC Biological Fungicide; T-22^®^ HC Biological Fungicide; RootShield^®^ Home and Garden Biological Fungicide; Root Guardian; RootShield^®^ Seed Treatment; RootShield^®^ Seed Treatment Biological Fungicide; RootShield^®^ AG; RootShield^®^ AG Biological Fungicide)	Bio Works Inc., Victor, NY, USA	http://www.pesticideinfo.org (accessed on 7 February 2021) https://www3.epa.gov/pesticides/chem_search/ppls/068539-00010-20190430.pdf (accessed on 7 February 2021) https://mycocosm.jgi.doe.gov/TriharT22_1/TriharT22_1.info.html (accessed on 7 February 2021)
			T-22™ HC		http://www.pesticideinfo.org (accessed on 7 February 2021)
			Trianum G Biological Fungicide (Trianum Granules Biological Fungicide; Trianum G)	Koppert Biological Systems, Inc., Howell, MI, USA	http://www.pesticideinfo.org (accessed on 7 February 2021) https://www3.epa.gov/pesticides/chem_search/ppls/089635-00003-20151005.pdf (accessed on 7 February 2021)
			Trianum WG Biological Fungicide (Trianum P Biological Fungicide; Trianum P; Trianum Granules Biological Fungicide; T-22 WG Water Dispersible Granules-Biological Fungicide)		http://www.pesticideinfo.org (accessed on 7 February 2021) https://www3.epa.gov/pesticides/chem_search/ppls/089635-00002-20150930.pdf (accessed on 7 February 2021)
*T. atrobrunneum* (formerly: *T. harzianum Rifai*)	ITEM 908	PNRQ10000000	Trianum P	Koppert Biological Systems, Inc., Howell, MI, USA	https://sitem.herts.ac.uk/aeru/bpdb/Reports/2034.htm (accessed on 7 February 2021)
Virens clade of *Trichoderma*					
*T. virens*	G-41	ATCC 20609	BW240 G ^a^ (RootShield^®^ Plus Granules, RootShield^®^ Plus Granules Biological Fungicide, TurfShield^®^ PLUS G, TurfShield^®^ PLUS G Biological Fungicide, TurfShield^®^ PLUS Granules, TurfShield^®^ PL US Granules Biological Fungicide)	Bio Works Inc., Victor, NY, USA	http://www.pesticideinfo.org (accessed on 7 February 2021) https://www.atcc.org/products/20906 (accessed on 7 February 2021)
Viride clade of *Trichoderma*					
*T. asperellum*	T34	EU077228 EU077227	T34 Biocontrol (Asperello T34 BIOCONTROL)	Biobest Biocontrol Technologies, Barcelona, Spain	https://www.pesticideinfo.org/ https://www3.epa.gov/pesticides/chem_search/ppls/087301-00001-20200403.pdf (accessed on 7 December 2021)
			Trifender Pro	Kwizda Agro Hungary Kft., Budapest, Hungary	https://sitem.herts.ac.uk/aeru/bpdb/Reports/2043.htm (accessed on 07 December 2021) https://kwizda.hu/AGRO_HU/products/t/Trifender%20Pro/Product%20Management/Registration%20Report/Trifender%20Pro%20Okirat.pdf (accessed on 7 December 2021)
	ICC012	GQ351595 GQ351596	Bioten ™ WP ^b^ (Tenet™ WP and Remedier WP and Tenet™ T&O)	Isagro USA, Inc., Morrisville, NC, USA	https://sitem.herts.ac.uk/aeru/bpdb/Reports/2043.htm (accessed on 7 December 2021) https://www3.epa.gov/pesticides/chem_search/ppls/080289-00009-20110217.pdf (accessed on 7 December 2021)
*T. atroviride*	I-1237		Esquive WP	Agrauxine, Marcq en Baroeul, France	https://sitem.herts.ac.uk/aeru/bpdb/Reports/2046.htm (accessed on 7 December 2021) https://www.efsa.europa.eu/en/efsajournal/pub/2706 (accessed on 7 December 2021)
			Tri-Soil	Agrauxine, Marcq en Baroeul, France	https://ephy.anses.fr/ppp/tri-soil (accessed on 7 December 2021) https://sitem.herts.ac.uk/aeru/bpdb (accessed on 7 December 2021)
	SC1	HV500891-500896	VINTEC^®^	Bi-PA nv, Londerzeel, Belgium	https://www.pesticideinfo.org/ https://sitem.herts.ac.uk/aeru/bpdb/Reports/2799.htm (accessed on 7 December 2021) https://www3.epa.gov/pesticides/chem_search/ppls/092083-00002-20200805.pdf (accessed on 7 December 2021)
*T. gamsii* (formerly: *T. viride*)	ICC080	GQ351598	Bioten ™ WP ^b^ (Tenet™ WP and Remedier WP and Tenet™ T&O)	Isagro USA, Inc., Morrisville, NC, USA	http://www.pesticideinfo.org (accessed on 7 December 2021) https://www3.epa.gov/pesticides (accessed on 7 December 2021)

^a^ Contains both *Trichoderma afroharzianum* Rifai strain T-22 and *Trichoderma virens* strain G-41; ^b^ Contains both *Trichoderma asperellum* ICC 012 and *Trichoderma gamsii* ICC 080.

**Table 2:** Molecular markers and annealing temperature used for the PCR amplification of *Trichoderma* sp.

Amplified Region	Primer	Reference	Annealing Temperature (°C)
ITS1/ITS2	SR6R	White et al. [36]	50
	LR1		
*tef1*	EF1 728F	Carbone and Kohn [37]	59
	EF1 986R		

**Table 3:** Plant pathogenic fungi used in the determination of the biocontrol activity of the *Trichoderma* strains.

Reference Number *	Fungal Pathogen	Host	Accession Number **
CBS 477.71	*Aphanomyces cochlioides*	*Beta vulgaris* L.	HQ665241
JT2015	*Botryosphaeria dothidea*	*Juglans regia* L.	MN706192
J2034	*Diaporthe eres*	*Juglans regia* L.	MT111103
HUT01	*Diplodia seriata*	*Vitis vinifera* L.	KU377167
R.3	*Eutypa lata*	*Vitis vinifera* L.	OK178559
B.CS.5.4.20.1.B	*Neofusicoccum parvum*	*Vitis vinifera* L.	OK178560
CBS 337.29	*Pythium acantophoron*	*Ananas sativus* (L.) Merr.	HQ665212

* Reference number in the CBS, or in the strain collection of the Microbiological Laboratory of Food Science Institute, University of Debrecen, Hungary. ** Accession number of the ribosomal DNA region.

**Table 4:** Systemic fungicides used for the determination *Trichoderma* strains tolerance. Tested concentration means their final concentration in the potato dextrose agar medium.

Target	Pesticide	Active Ingredient	Tested Concentration of the Pesticide (mg/L or mL/L)
Downy mildew	Orvego	Ametoctradin	399
		Dimethomorph	299.25
Powdery mildew	Rally Q SC	Myclobutanil,	45
		Quinoxifen	45
	Sercadis	Fluxapyroxad	225
	Talentum 20 EW	Myclobutanil	80
Grey mold	Chorus 50 WG	Cyprodinil	469
	Teldor 500 SC	Fenhexamid	835

**Table 5:** *Trichoderma* strains from endophytic woody tissues of ‘Furmint’ grapevine from the Tokaj Wine Region, Hungary in 2014.

*Trichoderma* Species	Strain No.	NCBI GenBank Accession No.	
		ITS ^a^	*tef1* ^b^
Harzianum clade			
*T. afroharzianum*	TR04	OK560827	OK655888
*T. atrobrunneum*	TR09	OK560832	OK655893
*T. harzianum*	TR07	OK560830	OK655891
	TR10	OK560833	OK655894
*T. simmonsii*	TR01	OK560824	OK655885
	TR02	OK560825	OK655886
	TR03	OK560826	OK655887
	TR05	OK560828	OK655889
Longibrachiatum Clade			
*T. orientale*	TR06	OK560829	OK655890
Viride Clade			
*T. gamsii*	TR08	OK560831	OK655892

^a^ ITS: Internal Transcribed Spacer. ^b^ *tef1*: Translation elongation factor 1-α.

**Table 6:** (**a**) Mycelial growth of endophytic *Trichoderma* strains from the Tokaj Wine Region, Hungary at 5 °C on potato dextrose agar (PDA) at 192 h and 336 h. Standard error (SE) is in separate column. Different letters show significant differences between species and strains based on pairwise analysis with Mann-Whitney U test (*p* < 0.05); (**b**) Mycelial growth of endophytic *Trichoderma* strains from the Tokaj Wine Region, Hungary at room temperature (18.5–25 °C) on PDA at 24 h and 54 h. Standard error (SE) is in separate column. Different letters show significant differences between species and strains based on pairwise analysis with Mann-Whitney U test (*p* < 0.05); (**c**) Mycelial growth of endophytic *Trichoderma* strains from the Tokaj Wine Region, Hungary at 37 °C on PDA at 24 h and 30 h. Standard error (SE) is in separate column. Different letters show significant differences between species and strains based on pairwise analysis with Mann-Whitney U test (*p* < 0.05).

**(a)**							
		**192 h**			**336 h**		
**Strains**	**Species**	**Mean**	**SE**		**Mean**	**SE**	
	Harzianum Clade						
	*T. afroharzianum*	16.17	2.20	ab	34.00	6.00	ab
	*T. atrobrunneum*	17.00	1.44	a	27.50	1.61	a
	*T. harzianum*	7.83	0.88	c	17.58	1.35	d
	*T. simmonsii*	14.92	0.64	ab	28.79	0.85	a
	Longibrachiatum Clade						
	*T. orientale*	12.67	0.17	b	22.67	0.44	b
	Viride Clade						
	*T. gamsii*	8.00	1.15	c	22.17	0.60	bc
TR01	*T. simmonsii*	15.33	0.44	a	30.33	0.93	a
TR02	*T. simmonsii*	17.83	1.09	b	31.17	0.60	a
TR03	*T. simmonsii*	13.83	0.33	a	29.17	0.60	a
TR05	*T. simmonsii*	12.67	0.17	c	24.50	1.04	b
TR07	*T. harzianum*	9.50	0.76	a	19.67	0.83	a
TR10	*T. harzianum*	6.17	0.73	b	15.50	2.02	a
**(b)**							
		**24 h**			**54 h**		
**Strains**	**Species**	**Mean**	**SE**		**Mean**	**SE**	
	Harzianum Clade						
	*T. afroharzianum*	17.06	0.87	b	56.42	3.81	ab
	*T. atrobrunneum*	18.29	0.41	b	51.25	2.42	a
	*T. harzianum*	13.85	0.49	a	47.55	2.31	a
	*T. simmonsii*	18.72	0.39	bc	60.13	2.04	bc
	Longibrachiatum Clade						
	*T. orientale*	26.00	0.80	d	72.17	3.65	d
	Viride Clade						
	*T. gamsii*	20.50	0.83	c	66.04	3.41	c
TR01	*T. simmonsii*	17.92	1.58		0.00	56.25	
TR02	*T. simmonsii*	18.58	1.59		0.00	57.54	
TR03	*T. simmonsii*	20.42	3.29		0.00	59.46	
TR05	*T. simmonsii*	17.96	3.34		0.00	67.25	
TR07	*T. harzianum*	14.13	2.11		0.00	47.79	
TR10	*T. harzianum*	13.58	2.76		0.00	47.31	
**(c)**							
		**24 h**			**30 h**		
**Strains**	**Species**	**Mean**	**SE**		**Mean**	**SE**	
	Harzianum Clade						
	*T. afroharzianum*	21.50	0.29	c	32.17	0.67	c
	*T. atrobrunneum*	13.17	0.17	b	16.17	0.17	b
	*T. harzianum*	14.17	0.53	b	18.33	0.44	b
	*T. simmonsii*	20.63	0.43	c	28.83	0.55	c
	Longibrachiatum Clade						
	*T. orientale*	39.83	0.33	d	58.67	0.17	d
	Viride Clade						
	*T. gamsii*	0.00	0.00	a	0.00	0.00	a
TR01	*T. simmonsii*	19.00	0.76		28.67	0.73	
TR02	*T. simmonsii*	21.83	0.67		30.00	0.29	
TR03	*T. simmonsii*	21.17	0.88		27.50	2.00	
TR05	*T. simmonsii*	20.50	0.29		29.17	0.67	
TR07	*T. harzianum*	14.00	1.00		18.50	0.87	
TR10	*T. harzianum*	14.33	0.60		18.17	0.44	

**Table 7:** Biocontrol activity expressed in biocontrol index (BCI) of the *Trichoderma* strains toward plant pathogen fungi. Standard deviation (SD) is in brackets.

	BCI (%)	
Fungal Pathogen	TR04	TR05
**Oomycota**		
*Aphanomyces cochlioides*	90.37 (0.64)	84.81 (0.64)
*Pythium acantophoron*	100.00 (0.00)	100.00 (0.00)
**Ascomycota**		
*Botryosphaeria dothidea*	100.00 (0.00)	25.19 (0.64)
*Diaporthe eres*	100.00 (0.00)	100.00 (0.00)
*Diplodia seriata*	100.00 (0.00)	100.00 (0.00)
*Eutypa lata*	100.00 (0.00)	100.00 (0.00)
*Neofusicoccum parvum*	95.19 (1.28)	90.00 (1.11)

**Table 8:** Mycelial growth inhibition of TR04 and TR05 *Trichoderma* strains by different systemic fungicides. Standard deviation (SD) is in brackets.

	Mycelial Growth Inhibition (%)	
Pesticide	TR04	TR05
Orvego	0.00 (0.00)	0.00 (0.00)
Rally Q SC	28.82 (1.78)	41.18 (2.10)
Sercadis	0.00 (0.00)	0.00 (0.00)
Talentum 20 EW	57.6 (2.44)	58.43 (0.61)
Chorus 50 WG	43.33 (0.48)	51.96 (0.98)
Teldor 500 SC	0.00 (0.00)	7.25 (1.88)

## Data Availability

The datasets used in the current study are available from the corresponding author on reasonable request.
